# Characterization of EPS subfractions from a mixed culture predominated by partial-denitrification functional bacteria

**DOI:** 10.1016/j.wroa.2024.100250

**Published:** 2024-08-27

**Authors:** Jiapeng Li, Yanxi Chen, Ji Qi, Xiaotian Zuo, Fangang Meng

**Affiliations:** a School of Environmental Science and Engineering, Sun Yat-sen University, Guangzhou 510275, PR China; b Guangdong Provincial Key Laboratory of Environmental Pollution Control and Remediation Technology (Sun Yat-sen University), Guangzhou 510275, PR China

**Keywords:** Extracellular polymeric substances, Partial denitrification (PD), Functional groups, Bacterial aggregation, Protein structure

## Abstract

Extracellular polymeric substances (EPS) play a crucial role in the aggregation of partial denitrification (PD) consortia, as EPS is closely linked to bioreactor performance. However, the structural and compositional properties of EPS from PD consortia have not yet been investigated. In this study, photometric measurements indicated that PD consortia contained significantly more EPS (168.81 ± 2.10 mg/g VSS) compared to conventional activated sludge (79.79 mg/g VSS). The EPS of PD consortia exhibited a significant predominance of proteins over polysaccharides, with a protein/polysaccharide ratio of 1.43 ± 0.10. FTIR analysis revealed that the EPS of PD consortia contained fewer hydrophilic functional groups, particularly carboxyl and carbonyl groups, indicating a high aggregation potential. The content comparison of EPS and functional groups across three stratified EPS subfractions from PD consortia consistently followed the sequence: TB-EPS > LB-EPS > S-EPS. XPS results corroborated the FTIR findings and the protein/polysaccharide ratio determined by photometric measurements, all of which suggested that the EPS of PD consortia exhibited a higher abundance of hydrophobic functional groups. However, the higher *α*-helix/(*β*-sheet + random coil) ratio (0.99) suggested that the proteins in PD consortia had a compact structure, making inner hydrophobic groups difficult to expose. This compact protein structure could limit aggregation among bacterial cells, indicating the need for process optimization to enhance sludge aggregation in PD-related processes. Overall, understanding the aggregation characteristics of PD consortia could improve the application of PD-based processes.

## Highlights

• The stratified EPS in PD consortia was characterized separately.
• PD consortia exhibited a higher EPS concentration than conventional activated sludge.
• The EPS of PD consortia contained higher abundance of hydrophobic functional groups.
• The compact protein structure limits the PD bacteria aggregation ability.

## Graphical abstract

**Figure:**
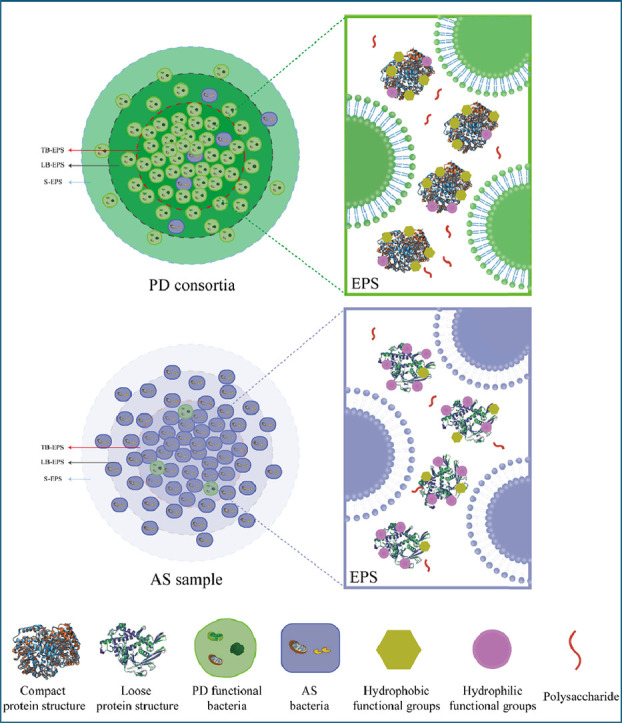

## 1 Introduction

Recent advancements in partial denitrification (PD) have garnered significant attention due to its potential to generate nitrite, which is crucial for achieving the anaerobic ammonia oxidation (anammox) process (Fan et al., 2022). The novel integration of PD and anammox processes offers substantial benefits, including at least a 50 % reduction in aeration demands, a 63 % reduction in carbon source requirements, and an 84 % reduction in sludge generation (Li et al., 2021; Zhang et al., 2024). Importantly, the enrichment and maintenance of PD and anammox functional bacteria are crucial for implementing the PD/anammox process at full scale. It is well known that increased cell density can significantly enhance microbial activity and promote growth rates (Freudenberg et al., 2021). The cell density is positively correlated with microbial aggregation ability, which is typically determined by the extracellular polymeric substances (EPS) secreted by bacteria (Jia et al., 2017).

EPS, an intricate matrix of high-molecular-weight biopolymers, is widely present in microbial aggregates (More et al., 2014). The characteristics of EPS largely determine the formation of bacterial aggregates and are strongly correlated with reactor performance (Ren et al., 2024). On one hand, EPS is amphoteric due to the coexistence of hydrophobic and hydrophilic groups, which influences the formation of microbial aggregates (Sheng et al., 2010). Enhanced hydrophobicity of the cell surface has been found to promote sludge aggregation and foster interactions among bacterial communities (Chao et al., 2014). On the other hand, EPS can serve as a medium facilitating the cross-feeding of nutrients and carbon sources within an environmental niche where denitrification and anammox bacteria cohabit and engage in symbiotic interactions (Guo et al., 2023; Zhang et al., 2021). Since the EPS is closely related to the interactions between PD bacteria and anammox bacteria, a deeper insight into the EPS characteristics of PD sludge aggregates is essential for achieving the successful coupling of PD and anammox.

To date, limited studies have investigated the EPS from PD consortia. For example, Fan et al. (2024) observed that the EPS content in PD sludge flocs increased from approximately 74.20 to nearly 112.90 mg/g VSS during the progressive startup and stabilization phases (Fan et al., 2024). Similarly, Lv et al. (2023) also found that the total EPS content increased with the achievement of PD (Lv et al., 2023). However, it should be noted that these studies focused solely on EPS content. The chemical and functional nature of EPS has not been well explored so far. In particular, a significant knowledge gap remains regarding the hydrophilic and hydrophobic characteristics of PD consortia. Protein hydrophobicity is largely determined by its secondary structure (Hou et al., 2015), yet previous efforts have rarely focused on the protein structural characteristics of PD consortia. Furthermore, the elemental composition of EPS from PD consortia also needs to be explored. Given these considerations, an in-depth understanding of the diverse characteristics of EPS in PD consortia is lacking.

To elucidate the EPS characteristics of PD consortia and shed light on their aggregation ability, we conducted an analysis of the composition and structural characteristics of EPS from PD consortia. In this study, the chemical compositions of EPS were monitored during PD operation. Additionally, 3D-EEM, FTIR, and XPS were extensively employed, accompanied by a comprehensive analysis of the amide I spectral range within EPS. Furthermore, a comparison of EPS between PD consortia and conventional sludge was conducted to reveal the unique nature of PD consortia. Finally, the implications of EPS characteristics from PD consortia for the optimization of PD and anammox processes were discussed.

## 2 Results and discussion

### 2.1 Variations in the chemical compositions of EPS subfractions

**Fig. 1:**
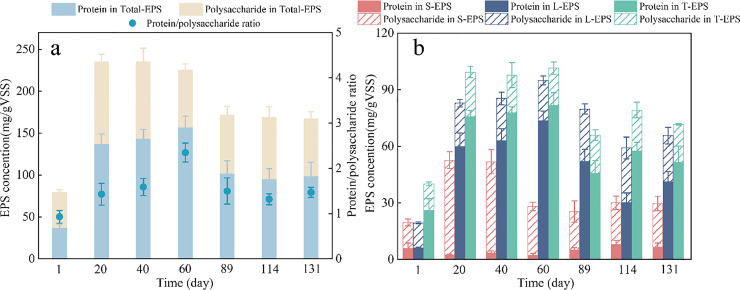
**The chemical compositions of EPS subfractions. (a)** Variations in the protein and polysaccharide concentrations as well as the protein/polysaccharide ratio of total EPS. **(b)** Variations in the proteins and polysaccharides of EPS subfractions.

Fig. 1 illustrates the variations in protein and polysaccharide content within EPS. The total contents of EPS subfractions increased rapidly during the start-up of PD (from 79.19 to 234.86 mg/g VSS), and then stabilized within a relatively consistent range (168.81 ± 2.10 mg/g VSS) (Fig. 1a). These findings are consistent with previous studies, which observed a gradual increase in EPS content during the start-up of PD (Fan et al., 2024; Lv et al., 2023). Among the three stratified EPS subfractions, tightly bound EPS (TB-EPS) exhibited the highest levels in most sludge samples, followed by loosely bound EPS (LB-EPS), with soluble EPS (S-EPS) consistently showing the lowest levels (Fig. 1b). Notably, the S-EPS of PD consortia contained more polysaccharides than proteins. Numerous studies have established that polysaccharides can form a strong and sticky framework, and the gelling properties arising from the network structure of polysaccharides could promote cell aggregation (Du et al., 2020; Sutherland, 2001). In contrast, the LB-EPS and TB-EPS of PD consortia contained more proteins than polysaccharides. Typically, proteins exhibit a stronger correlation with the surface properties of microbial aggregates, particularly hydrophobicity and surface charge, compared to polysaccharides (Hou et al., 2015; Liao et al., 2001). The high protein concentrations in LB-EPS and TB-EPS might promote the assembly and stabilization of microbial aggregates in PD consortia.

Furthermore, as indicated by the protein/polysaccharide ratios**,** the total EPS of PD consortia exhibited higher protein content than conventional activated sludge. The protein/polysaccharide ratio increased from 0.92 to 1.79 ± 0.49 and then stabilized at 1.43 ± 0.10. This suggests that proteins in EPS played a dominant role over polysaccharides during the start-up of PD. Previous studies have demonstrated that a higher protein/polysaccharide ratio is correlated with a less negatively charged surface and increased hydrophobicity (Zhang et al., 2007). It is well known that hydrophobic interactions among microbial cells are a critical mechanism contributing to cell aggregation (Neyens and Baeyens, 2003). This implies that the higher protein content in EPS from PD consortia might promote the aggregation of PD bacteria with other bacteria, such as anammox bacteria.

### 2.2 Three-dimensional Excitation-Emission Matrix (3D-EEM) fluorescence spectra analysis

**Fig. 2:**
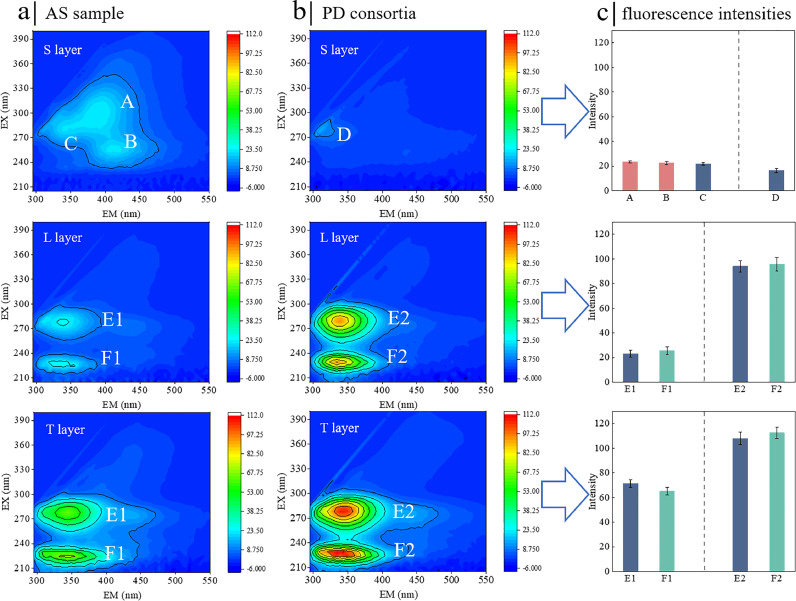
**The 3D-EEM fluorescence spectra of EPS. (a)** The spectra of EPS subfractions from AS sample. **(b)** The spectra of EPS subfractions from PD consortia. **(c)** The fluorescence intensities of typical peaks.

As illustrated in Fig. 2, three primary fluorescent constituents are identified: humic acid-like (peaks A and B), tryptophan protein-like (peaks C, D, and E), and aromatic protein-like substances (peak F).(Chen et al., 2003). In the S-EPS of conventional AS, there are three characteristic peaks (i.e., peaks A, B, C), while in PD consortia, only one characteristic peak (i.e., peak A) is present for PD consortia. In LB-EPS and TB-EPS, peaks E and F are observed. The intensities of these two peaks in PD consortia are much stronger than those in the AS sample. The peak intensity results align with the concentrations of protein content presented in Fig. 1. These results suggest that the fluorescent chemical constituents of EPS change during the start-up and steady operation of PD. Specifically, the PD consortia contain more tryptophan-like and aromatic protein-like substances but fewer humic acid-like substances. Notably, tryptophan-like and aromatic protein-like substances are expected to play significant roles in microbial aggregation and in maintaining structural robustness, whereas humic acid-like substances negatively impact the formation of sludge granules (Wilen et al., 2003). Thus, it can be expected that PD consortia is of better aggregation ability than conventional AS. Overall, the proteins, polysaccharides, and 3D-EEM fluorescence spectra show that PD consortia contain more EPS than conventional AS. Therefore, we hypothesize that the abundance of functional groups may also be higher in the PD consortia. This hypothesis is later verified by the Fourier transform infrared (FTIR) results.

### 2.3 FTIR spectroscopy analysis

**Fig. 3:**
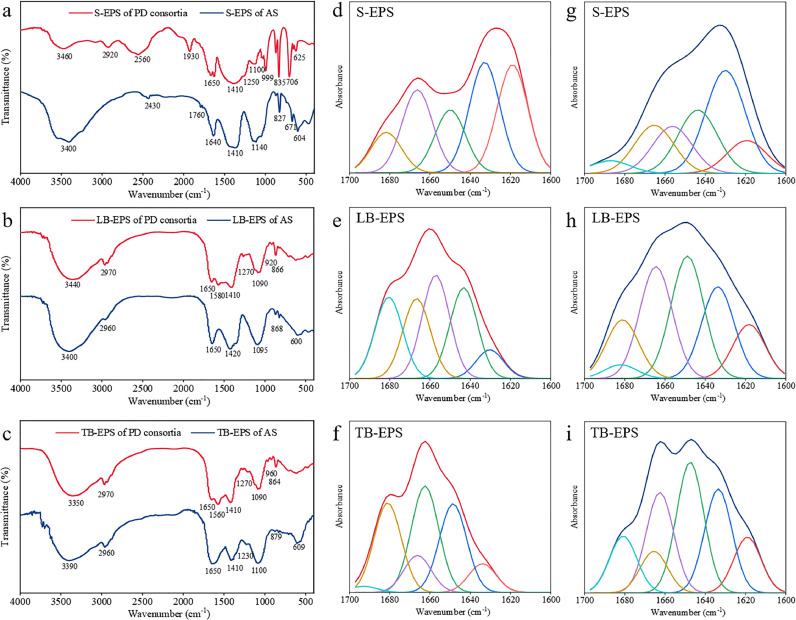
**FTIR spectra of EPS. (a–c)** FTIR spectra (4000–400 cm^−1^) of EPS subfractions from AS and PD consortia. **(d-f)** The second derivative resolution-enhanced curve-fitted amide I region (1700–1600 cm^−1^) for protein from EPS subfractions of PD consortia. **(g–i)** The second derivative resolution-enhanced curve-fitted amide I region (1700–1600 cm^−1^) for protein from the EPS subfractions of AS.

FTIR spectroscopy analysis was conducted to examine the functional groups of EPS across the 4000-400 cm^−1^ spectral range (Fig. 3). The functional groups of EPS, consistent with previous studies on FTIR spectral features (Badireddy et al., 2008; Sun et al., 2023), primarily correspond to carbohydrates, proteins, polysaccharides, and nucleic acids (Supplementary Table S1-2). The transmittance rates of EPS subfractions from PD consortia followed the sequence of S-EPS > LB-EPS > TB-EPS, suggesting that the abundance of functional groups among the subfractions was in the order of S-EPS < LB-EPS < TB-EPS. This result aligns with the photometric measurements (Fig. 1). The spectral region spanning 1800–400 cm^−1^ is known to provide crucial information on the composition and functional characteristics of EPS constituents (Hou et al., 2022; Yin et al., 2015). Although the EPS subfractions of PD consortia exhibited comparable peak positions to those observed in the AS sample, a detailed analysis of peak intensities reveals notable differences in the relative abundance of chemical groups: (i) The spectral peak around 1650 cm^−1^, attributed to C=O stretching vibrations of peptide groups, was detected in the EPS subfractions of both AS and PD consortia. This peak was noticeably smaller in PD consortia than in the AS sample. (ii) The peak of the amide II band (1600–1500 cm^−1^) attributed to N-H bending and C-N stretching vibrations, was absent in the AS sample and notably weak in PD consortia. (iii) The band around 1410 cm^−1^ indicative of the symmetric stretching of C=O in COO⁻, was also much smaller for PD consortia than the AS sample. (iv) The amide III band (1300–1200 cm^−1^), characterized by C-N stretching associated with secondary amides of proteins, was weak for both sludge samples. These results indicate a lower abundance of hydrophilic functional groups (e.g., carboxyl and carbonyl groups) in the EPS from PD consortia, providing insight into the hydrophobic nature of EPS from PD consortia. Additionally, a prominent peak around 1100 cm^−1^ signifies the presence of polysaccharides, indicating their significant contribution to EPS. This peak is also noticeably smaller in PD consortia than in the AS sample, further supporting the higher polysaccharide/protein ratio in the EPS from AS.

In light of the critical role that protein secondary structures play in the aggregation potential of bacterial cells (Wang et al., 2020), the secondary structure assignments of proteins were determined using spectral data obtained from reference proteins and peptides with confirmed structural configurations (Beech et al., 1999). The corresponding protein secondary structures for EPS from PD consortia are outlined in Table 1. Among these, aggregated strands and *β*-sheets were predominantly abundant in the S-EPS, while the random coil was only detectable in the LB-EPS. Within the amide I spectral range, 3-turn helices were predominant compared to other protein secondary structures, particularly prevalent in TB-EPS (45.75 %). The distribution of antiparallel *β*-sheet or aggregate strand functional groups varied among EPS layers, following the order of TB-EPS > LB-EPS > S-EPS. Previous studies have reported that the *α*-helix/(*β*-sheet + random coil) ratio can serve as an indicator of protein structure, with lower values signifying a less compact protein structure (Dsane et al., 2023; Yang et al., 2024). Proteins with a less compact structure are thought to enhance the accessibility of internal hydrophobic functional groups (Wang and Wang, 2023). The *α*-helix/(*β*-sheet + random coil) values for the three stratified EPS subfractions of PD consortia were determined to be 0.57, 0.86, and 3.09, respectively. The marked differences in these values suggest distinct protein secondary structures among the EPS layers. Compared to the lower values of 0.28, 0, and 1.33 found in the AS sample, the relatively higher *α*-helix/(*β*-sheet + random coil) values in the EPS subfractions of PD consortia suggest a more compact protein structure, which can enclose hydrophobic functional groups and diminish hydrophobic interactions. Furthermore, the *α*-helix/(*β*-sheet + random coil) ratio of total EPS (0.99) is also notably higher compared to other types of sludge previously reported (Hou et al., 2015; Wang et al., 2020), e.g., 0.3 (anammox sludge), 0.51 (activated sludge), 0.46 (nitrifying sludge), and 0.44 (denitrifying sludge). These results indicate that, although more hydrophobic groups are present in EPS from PD consortia, the proteins in the PD consortia exhibit a more compact structure, resulting in reduced exposure of inner hydrophobic groups and thereby limiting the expression of hydrophobic properties. This compact structure is not conducive to the aggregation of PD bacteria with other bacteria.

**Table 1:** Distribution of secondary structure types among EPS proteins from PD consortia.

Secondary structures	Wavenumber (cm^−1^)	At %			
		S-EPS	LB-EPS	TB-EPS	Total EPS
Aggregated strands	1625-1610	26.50 %	0.14 %	-	8.88 %
*β*-sheet	1640-1630	27.11 %	7.46 %	7.30 %	13.96 %
Random coil	1645-1640	-	23.60 %	-	7.87 %
*α*-Helix	1657-1648	15.40 %	26.86 %	22.58 %	21.61 %
3-Turn helix	1666-1659	20.35 %	20.76 %	45.75 %	28.95 %
Antiparallel *β*-sheet or Aggregate strands	1695-1680	10.63 %	21.18 %	24.37 %	18.73 %
*α*-helix/(*β*-sheet + random coil)	-	0.57	0.86	3.09	0.99

### 2.4 x-ray photoelectron spectroscopy (XPS) analysis

**Fig. 4:**
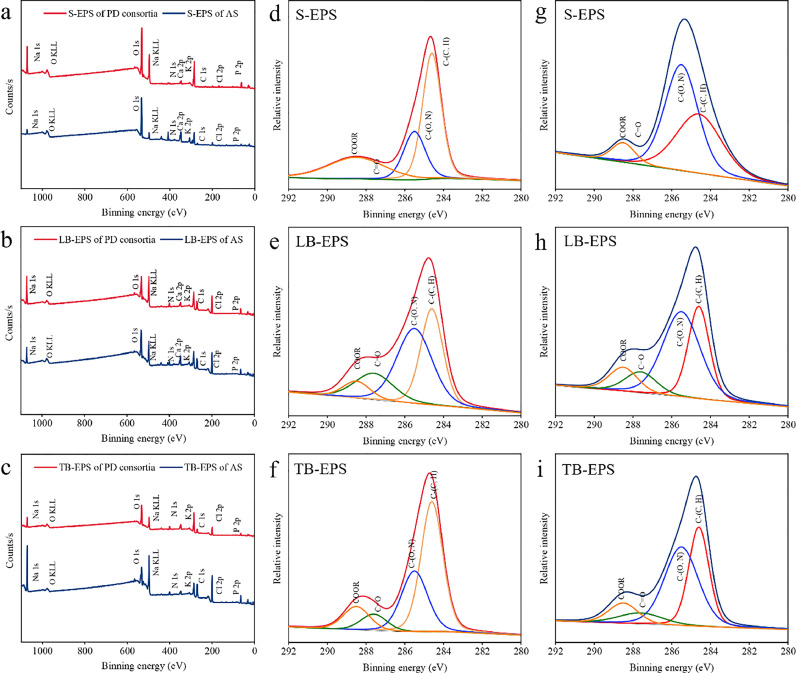
**XPS spectra of EPS. (a–c)** XPS wide survey scans of the EPS subfractions from AS and PD consortia. **(d–f)** High-resolution XPS data for C 1s in the EPS subfractions from PD consortia. **(g–i)** High-resolution XPS data for C 1s in the EPS subfractions from AS sample.

Elemental analysis of the EPS samples was performed using XPS within the energy spectrum of 0–1100 eV (Fig. 4). The elemental compositions of C, O, and N in EPS subfractions from AS and PD consortia are shown in Table 2. The EPS from PD consortia exhibited lower O/C (0.56) and N/C (0.09) ratios than EPS from AS (0.95 for O/C and 0.24 for N/C). Carbon was found to be the major constituent in the EPS from PD consortia.

**Table 2:** The elemental ratios of N and O, as well as the functional groups relative to C determined from the high-resolution XPS spectra.

EPS	Layers	Elemental composition (expressed as molar ratios relative to total carbon content)		Chemical functionalities (expressed as molar ratios relative to total carbon)								
		O/C	N/C	284.6 eV C-(C,H)	285.5 eV C-(O,N)	287.6 eV C=O	288.5 eV COOR	531.4 eV O=C	532.7 eV C-OH, C-O-C	398 eV NSi_3_	399.42 eV C-NH_2_	399.9 eV N_nonpr_
AS	S-EPS	1.78	0.42	0.404	0.528	0.000	0.068	0.388	1.392	0.033	0.122	0.265
	LB-EPS	0.63	0.17	0.303	0.488	0.103	0.106	0.578	0.052	0.024	0.061	0.085
	TB-EPS	0.45	0.12	0.349	0.462	0.086	0.103	0.326	0.124	0.005	0.061	0.054
	Total	0.95	0.24	0.352	0.493	0.063	0.092	0.589	0.361	0.021	0.093	0.126
PD	S-EPS	0.85	0.05	0.509	0.225	0.000	0.266	0.545	0.305	0.003	0.043	0.003
	LB-EPS	0.48	0.13	0.330	0.334	0.245	0.091	0.447	0.033	0.023	0.043	0.064
	TB-EPS	0.36	0.08	0.517	0.291	0.079	0.113	0.235	0.125	0.004	0.028	0.048
	Total	0.56	0.09	0.464	0.292	0.143	0.101	0.416	0.144	0.009	0.047	0.035

To acquire a comprehensive understanding of the EPS chemical properties, the XPS spectra for C 1s, O 1s, and N 1s were subjected to high-resolution deconvolution (Table 2). The C 1s spectrum was deconvoluted into four distinct components: C-(C, H), C-(O, N), C=O, and COOR (Fig. 4d-i). The component at 284.6 eV was identified as C-(C, H), primarily associated with lipid or amino acid side chains. This functional group exhibited higher prevalence in the total EPS from PD consortia, comprising 46.4 % of the total C 1s content compared to EPS from AS (35.2 %), and was predominantly found in TB-EPS (51.7 %). The component at 285.5 eV corresponds to C-(O, N) groups, which are indicative of alcohol, ether, amine, or amide groups within proteins. In contrast, this functional group was much higher in the total EPS from AS (49.3 %) than in that from PD consortia (29.2 %). The weak peaks at 287.6 eV and 288.5 eV were attributable to C=O and COOR, respectively. Among the four components, C-(C, H) is associated with the hydrophobic characteristics of EPS, whereas the remaining three components indicate its hydrophilic nature. This observation aligns with the protein/polysaccharide ratio and FTIR results, further indicating a higher prevalence of hydrophobic functional groups in the EPS from PD consortia.

The broad O 1s spectrum was resolved into three distinct components (Supplementary Fig. S2). The spectral feature around 531.5 eV is primarily associated with O=C groups, including carboxylate, carbonyl, ester, or amide functionalities, contributing to molar ratios of 58.9 % and 41.6 % of the total carbon content in EPS obtained from AS and PD consortia, respectively. The secondary peak at 532.5 eV is attributed to O-(C, H) groups, which are associated with acetal, hemiacetal, or alcohol functionalities (Badireddy et al., 2008). The proportion of O-(C, H) groups in the total EPS extracted from AS reached 36.1 %, compared to only 14.4 % in the EPS extracted from PD consortia (Table 2). The N 1s spectrum was also deconvoluted into three distinct components (Supplementary Fig. S3). The spectral feature at 399.9 eV is assigned to non-protonated nitrogen (N_nonpr_), while the peaks at 398 eV and 399.42 eV originate from NSi_3_ and C-NH_2_.

### 2.5 Implication of this study

Identifying the key components of EPS is crucial for understanding the characteristics of PD bacteria and for optimizing the performance of reactors with PD and anammox processes. In this study, we found that the contents and components of EPS varied significantly during the start-up and steady operation of the PD process. The EPS extracted from PD consortia exhibited significant differences compared to those extracted from AS samples. Firstly, the total content of the EPS subfractions increased rapidly during the start-up of PD. Additionally, the EPS of PD consortia comprised more proteins than those of AS samples, as determined by the protein/polysaccharide ratios. Secondly, tryptophan-like and aromatic protein-like substances were enriched in the EPS from PD consortia, particularly in TB-EPS, compared to the EPS from AS. Thirdly, the FTIR and XPS spectra indicated a much lower abundance of hydrophilic groups in the EPS from PD consortia, particularly C=O, COO-, and C-(O, N) groups. These findings elucidate the hydrophobic characteristics of EPS from PD consortia. Therefore, we can conclude that the PD consortia have a high aggregation potential. However, the higher *α*-helix/(*β*-sheet + random coil) value of PD consortia indicates that the proteins possess a more compact structure, thereby reducing the exposure of internal hydrophobic groups. The presence of compact protein structures might be a critical limitation for PD bacteria aggregation with other functional bacteria.

The novel integration of PD and anammox processes exhibits substantial technical advantages, such as low aeration costs and reduced carbon source usage. However, there are many challenges due to the vulnerability of anammox bacteria to environmental stresses. Notably, the sludge aggregation ability of the PD system is a potential determinant for the enrichment of anammox bacteria. Studies have demonstrated that carbon source types, dissolved oxygen, salinity, and alkalinity (Li et al., 2024; Liu et al., 2021; Suresh et al., 2019; Wang et al., 2013) can influence the secretion and properties of proteins. Additionally, shear rate, pH, and temperature (Chen et al., 2024; Menniti et al., 2009; Sweity et al., 2011) impact polysaccharide secretion by cells. Thus, selecting appropriate process parameters can regulate sludge aggregation. Overall, this study clarifies the EPS characteristics of PD consortia, which will facilitate the advancement of PD and anammox process applications.

## 3 Conclusions

The primary conclusions of this study are outlined below:

(1) The PD consortia contained more EPS compared to conventional AS, with the EPS in PD consortia showing a significant predominance of proteins over polysaccharides.
(2) The EPS from PD consortia contained fewer hydrophilic functional groups, particularly carboxyl and carbonyl groups, and demonstrated a high aggregation potential. XPS results corroborated the FTIR findings and the protein/polysaccharide ratio, further suggesting that the EPS of PD consortia had a higher abundance of hydrophobic functional groups.
(3) The higher *α*-helix/(*β*-sheet + random coil) value indicated that the proteins in the EPS from PD consortia had a more compact structure, making the inner hydrophobic groups less accessible. This compact protein structure affects the ability of PD bacteria to aggregate with other functional bacteria.

## 4 Materials and methods

### 4.1 Microorganism and bioreactor operation

The sludge inoculum was obtained from a wastewater treatment facility in Zhuhai City. A laboratory-scale reactor was operated for a prolonged period of 100 days, characterized by a sequencing batch operational mode. This reactor maintained a hydraulic retention time of 4 h and a sludge retention time of 20–25 days during operation, with influent NO_3_^−^-N and COD/N concentrations of 33.45 ± 1.05 mg N/L and 4.4 ± 0.2, respectively. The long-term operational performance and microbial community structure of the reactor are described in the supplementary materials. Notably, the sludge was sampled for EPS analysis at the beginning of operation and on the 60th day.

### 4.2 EPS extraction and analysis

An optimized thermal extraction technique for EPS, following the procedures outlined by Li and Zhao (Li and Yang, 2007; Zhao et al., 2016), was used to extract S-EPS, LB-EPS, and TB-EPS. Detailed extraction protocols can be found in the supplementary materials. Protein and polysaccharide concentrations in EPS samples from each layer were quantified using the Lowry-Folin and phenol-sulfuric acid methods, respectively (Frolund et al., 1995; Lowry et al., 1951; McKnight, 1977). The total content of EPS was determined by summing the protein and polysaccharide levels. Additionally, to further characterize EPS components, samples from each layer were analyzed using 3D-EEM spectroscopy. The 3D-EEM spectra were recorded with a fluorescence spectrometer (HITACHI, F-4700) following the approach detailed by (Miao et al., 2016).

### 4.3 FTIR and XPS analysis

An FTIR spectrometer from Bruker (Germany) was used to analyze the functional groups within EPS subfractions. Samples were first freeze-dried and mixed with KBr powder suitable for FTIR analysis. The resulting spectra were generated and processed using OPUS (v5.0) software. To determine the elemental composition and functional groups, an Axis Ultra XPS system from Thermo Fisher Scientific (UK) was employed. A broad survey scan with a pass energy of 20.0 eV was conducted to identify the principal elemental constituents, followed by a detailed high-resolution scan at pass energy of 100.0 eV to explore component speciation.

## CRediT authorship contribution statement

**Jiapeng Li:** Writing – review & editing, Writing – original draft, Supervision, Methodology. **Yanxi Chen:** Methodology, Investigation. **Ji Qi:** Validation. **Xiaotian Zuo:** Supervision. **Fangang Meng:** Writing – review & editing, Supervision, Project administration, Funding acquisition.

## Declaration of competing interest

The authors declare the following financial interests/personal relationships which may be considered as potential competing interests:

Fangang Meng reports financial support was provided by Basic and Applied Basic Research Foundation of Guangdong Province. Fangang Meng reports financial support was provided by National Natural Science Foundation of China. Fangang Meng reports was provided by Fundamental Research Funds for the Central Universities. If there are other authors, they declare that they have no known competing financial interests or personal relationships that could have appeared to influence the work reported in this paper.

## Data Availability

Data will be made available on request.

## Appendix Supplementary materials

Image, application 1
