# Supplementary material for: Characterization of EPS subfractions from a mixed culture predominated by partial-denitrification functional bacteria
Source: Water Res X. 2024 Aug 27;24:100250. doi: 10.1016/j.wroa.2024.100250 (PMC11402163; doi:10.1016/j.wroa.2024.100250)
Supplement: Supplementary file 1 [file mmc1.docx]

**Supporting information for:**

**Characterization of EPS subfractions from a mixed culture predominated by partial-denitrification functional bacteria**

Jiapeng Li ^a,b^, Yanxi Chen ^a,b^, Ji Qi ^a,b^, Xiaotian Zuo ^a,b^, Fangang Meng ^a,b *^

^a^ School of Environmental Science and Engineering, Sun Yat-sen University, Guangzhou 510275, PR China

^b^ Guangdong Provincial Key Laboratory of Environmental Pollution Control and Remediation Technology (Sun Yat-sen University), Guangzhou 510275, PR China

*** Corresponding author**

Fangang Meng, Ph.D.

Email: [mengfg@mail.sysu.edu.cn](mailto:mengfg@mail.sysu.edu.cn)

Tel: 86-20-39335060

Fax: 86-20-84110267

Number of pages: 10

Number of methods: 1

Number of figures: 3

Number of texts: 1

Number of tables: 3

**Supplementary Methods**

**Method S1** The extraction methods for three stratified EPS subfractions.

**Supplementary Figures**

**Fig. S1.** Reactor performance and microbial community structure. (a) The variation of nitrate to nitrite ratio (NTR) in anoxic phase during the long-term operation. (b) The variation of specific NO_3_^-^-N reduction activity during the long-term operation. (c-d) The microbial community structure at the phylum and genus level on Day1 and Day95.

**Fig. S2.** XPS spectra of EPS. (a-c) High-resolution XPS data for O 1s in EPS subfractions from PD consortia. (d-f) High-resolution XPS data for O 1s in EPS subfractions from AS sample.

**Fig. S3.** XPS spectra of EPS. (a-c) High-resolution XPS data for N 1s in EPS subfractions from PD consortia. (d-f) High-resolution XPS data for N 1s in EPS subfractions from AS sample.

**Supplementary Texts**

**Text S1** The long-term operational performance and microbial community structure of the reactor.

**Supplementary Tables**

**Table S1** Band assignments for the FTIR spectral features (cm^-1^) of EPS from AS sample.

**Table S2** Band assignments for the FTIR spectral features (cm^-1^) of EPS from PD consortia.

**Table S3** Distribution of secondary structure types among EPS proteins from AS sample.

**Method S1 The extraction methods for three stratified EPS subfractions**

The samples were dispersed in tubes separately, and were diluted to 40 mL followed by centrifugation at 4000 g for 5 min. The supernatant liquid was filtered with 0.45 μm polytetrafluoroethylene filters and was analyzed as the soluble EPS (S-EPS). After removing the supernatant, the mixture was added with 0.05% NaCl solution to a final volume of 40 mL, and was vortexed for 1 min. The samples were centrifuged at 4000 g for 10 min, and the supernatant filtered through 0.45 μm polytetrafluoroethylene filters was analyzed as the loosely-bound EPS (LB-EPS). Subsequently, the 0.05% NaCl solution was added again to keep a volume of 40 mL. The sludge mixture was placed at 60 ℃ water bath for 30 min, and was shaken every 5 min followed by centrifugation at 4000 g for 15 min. The supernatant filtered through 0.45 μm polytetrafluoroethylene filters was analyzed as the tightly-bound EPS (TB-EPS).

**
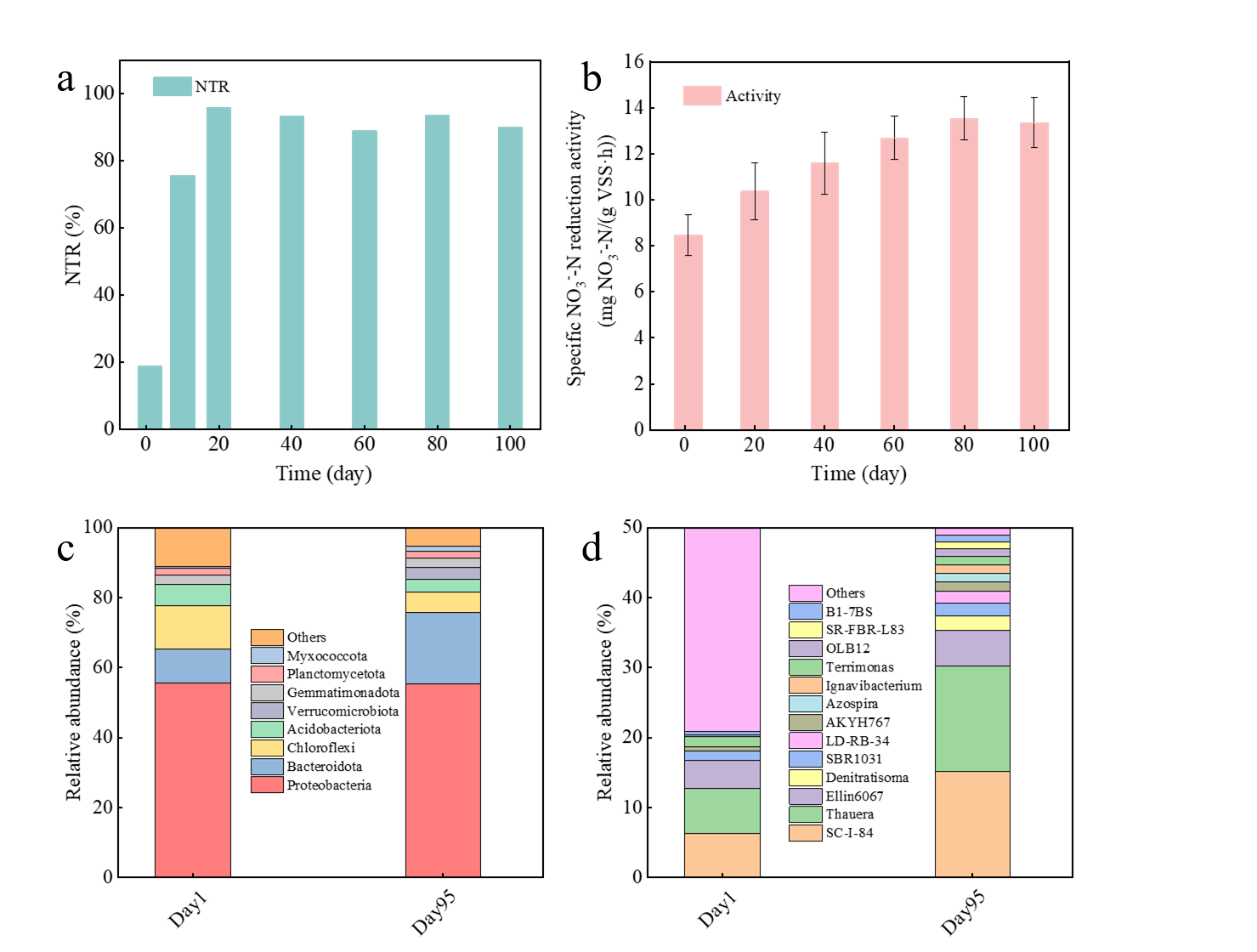
**

**Fig. S1. Reactor performance and microbial community structure. (a)** The variation of nitrate to nitrite ratio (NTR) in anoxic phase during the long-term operation. **(b)** The variation of specific NO_3_^-^-N reduction activity during the long-term operation. **(c-d)** The microbial community structure at the phylum and genus level on Day1 and Day95.

**
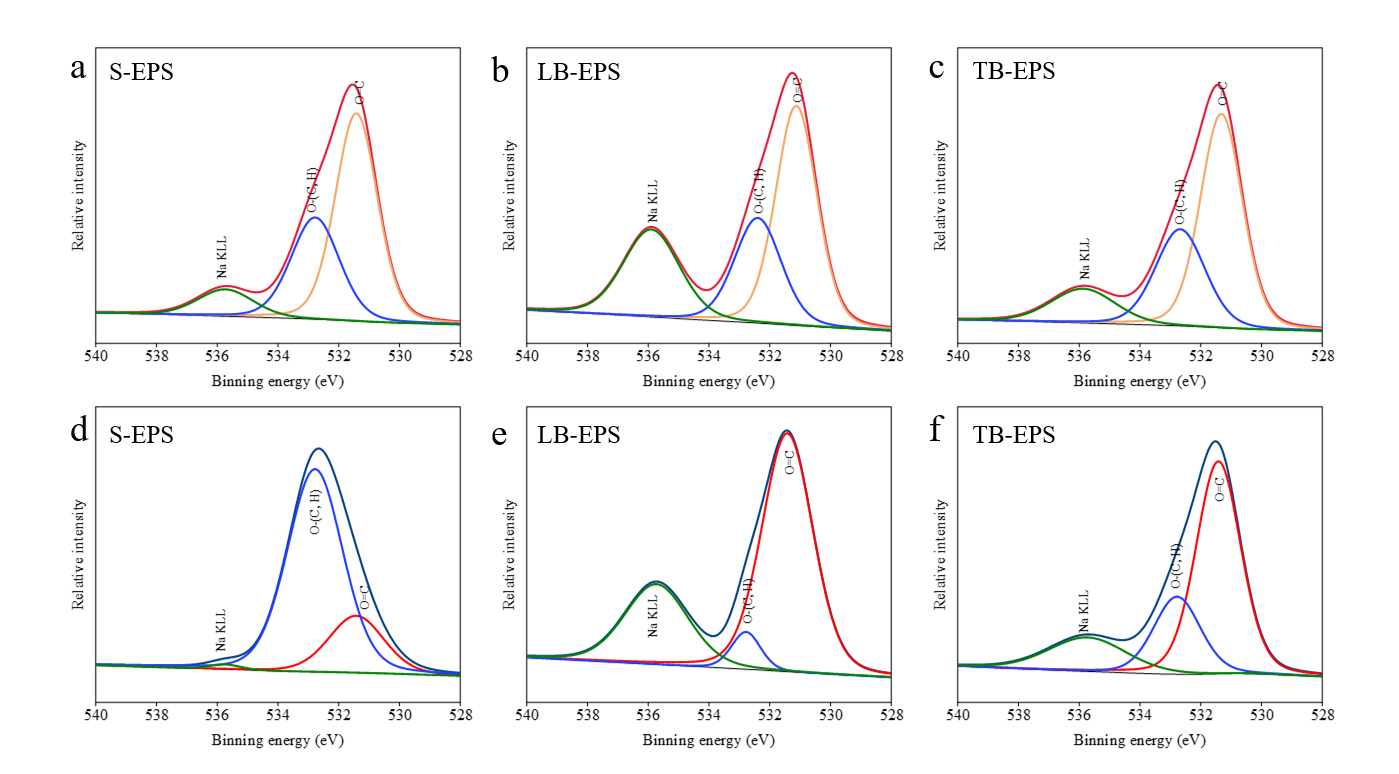
**

**Fig. S2. XPS spectra of EPS.** **(a-c)** High-resolution XPS data for O 1s in EPS subfractions from PD consortia. **(d-f)** High-resolution XPS data for O 1s in EPS subfractions from AS sample.

**
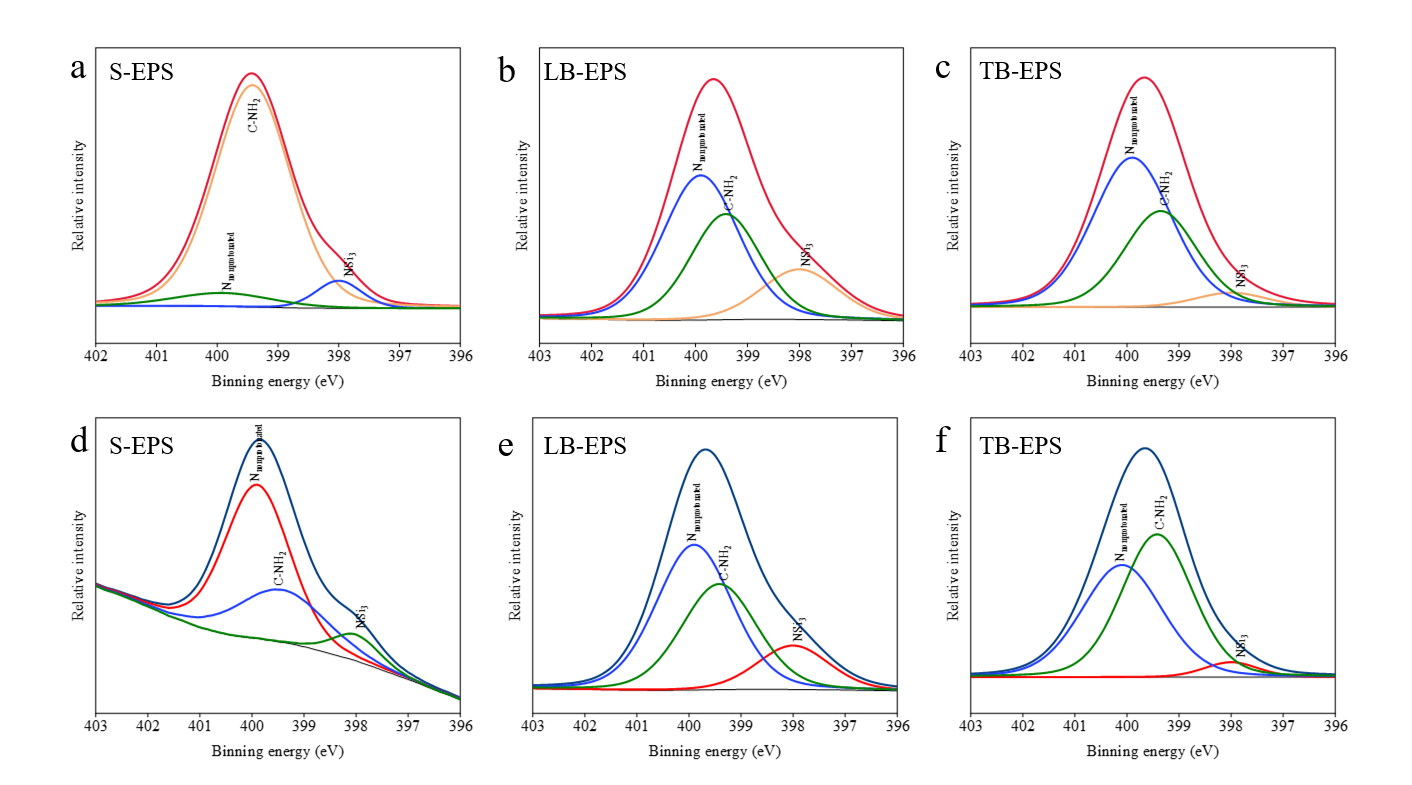
**

**Fig. S3. XPS spectra of EPS.** **(a-c)** High-resolution XPS data for N 1s in EPS subfractions from PD consortia. **(d-f)** High-resolution XPS data for N 1s in EPS subfractions from AS sample.

**Text S1 The long-term operational performance and microbial community structure of the reactor**

The long-term operational performance and microbial community structure of the reactor were depicted in Fig. S1. The nitrate to nitrite ratio (NTR) in anoxic phase escalated rapidly from 18.83% on the first day to 75.69% on the 10th day, indicative of the rapid start-up of PD. From the 20th day on, the NTR reached a peak of 95.81%, and maintained a relatively high level of 90.38 ± 4.65% throughout the extended 100-day operation. With the start-up of PD, *Proteobacteria*, *Bacteroidota*, *Chloroflexi*, and *Actinobacteriota* were always the dominant phyla. At the genus level, a significant increase in the abundance of *Thauera* and *SC-I-84* was observed. *Thauera* had already been acknowledged as a primary species in denitrification processes due to its high capacity for NO_2_^-^-N accumulation (Zhang et al., 2023). The genus *SC-I-84*, previously observed in systems with strong denitrification capabilities, likely contributed to the increasing denitrification capacity of the system, evidenced by the positive correlation between nitrite accumulation rate and the abundance of this genus (Sun and Zhu, 2022).

| **Table S1 Band assignments for the FTIR spectral features (cm^-1^) of EPS from AS sample.** | | | | |
| --- | --- | --- | --- | --- |
| Secondary structures | Wavenumber (cm^-1^) | | | Band assignments |
|  | S-EPS | L-EPS | T-EPS |  |
| Hydrocarbons | 3400 | 3400 | 3390 | O-H stretching (hydrogen-bonded) |
|  | - | 2960 | 2960 | C-H stretching (CH_2_ and CH_3_groups) |
| Proteins | 1640 | 1650 | 1650 | C=O stretch (amide I) associated with proteins |
|  | 1410 | 1420 | 1410 | C=O symmetric stretching of -COO- groups (amide II) |
| Polysaccharides | 1140 | 1090 | 1080 | C-OH and C-O possibly associated with polysaccharide |
| Nucleic acids | 827 | 868 | 879 | asymmetric ester O-P-O stretching modes from nucleic acids |

| **Table S2 Band assignments for the FTIR spectral features (cm^-1^) of EPS from PD consortia.** | | | | |
| --- | --- | --- | --- | --- |
| Region | Wavenumber (cm^-1^) | | | Band assignments |
|  | S-EPS | LB-EPS | TB-EPS |  |
| Hydrocarbons | 3460 | 3440 | 3350 | O-H stretching (hydrogen-bonded) |
|  | 2920 | 2970 | 2970 | C-H stretching (CH_2_ and CH_3_ groups) |
| Proteins | 1650 | 1650 | 1650 | C=O stretch (amide I) associated with proteins |
|  | 1400 | 1410 | 1410 | C=O symmetric stretching of -COO- groups (amide II) |
|  | 1250 | 1270 | 1270 | C-N stretching associated with secondary amides of proteins (amide III) |
| Polysaccharides | 1100 | 1080 | 1080 | C-OH and C-O possibly associated with polysaccharide |
| Nucleic acids | 999 | 920 | 960 | asymmetric ester O-P-O stretching modes from nucleic acids |

| **Table S3 Distribution of secondary structure types among EPS proteins from AS sample.** | | | | | |
| --- | --- | --- | --- | --- | --- |
| Secondary structures | Wavenumber (cm^-1^) | At % | | | |
|  |  | S-EPS | LB-EPS | TB-EPS | Total EPS |
| Aggregated strands | 1625-1610 | 10.87% | 12.81% | 11.65% | 11.78% |
| *β*-sheet | 1640-1630 | 34.07% | 23.86% | 19.78% | 25.90% |
| Random coil | 1645-1640 | 20.95% | - | - | 6.98% |
| *α*-Helix | 1657-1648 | 15.55% | - | 26.39% | 13.98% |
| 3-Turn helix | 1666-1659 | 14.37% | 50.27% | 24.13% | 29.59% |
| Antiparallel *β*-sheet or aggregate strands | 1695-1680 | 4.18% | 13.06% | 18.05% | 11.76% |
| *α*-helix/(*β*-sheet + random coil) | - | 0.28 | 0 | 1.33 | 0.43 |

**References**

Sun, Q. and Zhu, G. 2022. Deciphering the effects of antibiotics on nitrogen removal and bacterial communities of autotrophic denitrification systems in a three-dimensional biofilm electrode reactor. Environmental Pollution 315, 120476.

Zhang, M., Liu, J., Liang, J., Fan, Y., Gu, X. and Wu, J. 2023. Response of nitrite accumulation, sludge characteristic and microbial transition to carbon source during the partial denitrification (PD) process. Science of The Total Environment 894, 165043.
